# About the Reliability of CALPHAD Predictions in Multicomponent Systems

**DOI:** 10.3390/e20120899

**Published:** 2018-11-24

**Authors:** Stéphane Gorsse, Oleg N. Senkov

**Affiliations:** 1 CNRS, Université de Bordeaux, ICMCB, UMR 5026, F-33600 Pessac, France; 2 Bordeaux INP, ENSCBP, F-33600 Pessac, France; 3 Air Force Research Laboratory, Materials and Manufacturing Directorate, Wright-Patterson AFB, OH 45433, USA

**Keywords:** alloy design, structural metals, multi-principal element alloys, CALPHAD

## Abstract

This study examines one of the limitations of CALPHAD databases when applied to high entropy alloys and complex concentrated alloys. We estimate the level of the thermodynamic description, which is still sufficient to correctly predict thermodynamic properties of quaternary alloy systems, by comparing the results of CALPHAD calculations where quaternary phase space is extrapolated from binary descriptions to those resulting from complete binary and ternary interaction descriptions. Our analysis has shown that the thermodynamic properties of a quaternary alloy can be correctly predicted by direct extrapolation from the respective fully assessed binary systems (i.e., without ternary descriptions) only when (i) the binary miscibility gaps are not present, (ii) binary intermetallic phases are not present or present in a few quantities (i.e., when the system has low density of phase boundaries), and (iii) ternary intermetallic phases are not present. Because the locations of the phase boundaries and possibility of formation of ternary phases are not known when evaluating novel composition space, a higher credibility database is still preferable, while the calculations using lower credibility databases may be questionable and require additional experimental verification. We estimate the level of the thermodynamic description which would be still sufficient to correctly predict thermodynamic properties of quaternary alloy systems. The main factors affecting the accuracy of the thermodynamic predictions in quaternary alloys are identified by comparing the results of CALPHAD calculations where quaternary phase space is extrapolated from binary descriptions to those resulting from ternary system descriptions.

## 1. Introduction

Computational approaches are now a vital aspect of materials science, which enable the prediction of unknown data, such as materials properties and phase stability, and the more rapid exploration of design space. CALPHAD (Calculation of Phase Diagrams) is an efficient computational thermodynamic technique which permits the prediction of the phase equilibria and thermodynamic properties of multicomponent systems from those of the respective binary and ternary subsystems. CALPHAD practices also include the simulation of solidification, mapping of onset driving forces and nucleation barriers for precipitation [1], modeling of phase transformations [2], and estimation of stacking fault energy [3], for example. The development of multicomponent thermodynamic databases caused a growing interest in the calculations of phase diagrams for high entropy alloys (HEAs) and complex concentrated alloys (CCAs) [3,4,5,6,7] allowing the exploration of the composition space to be accelerated [8,9,10]. In this context, it is of paramount importance to analyze the reliability of the predictions made using commercial dedicated HEA databases.

The reliability of the predictions depends upon the quality of the assessment of the Gibbs energy parameters of the phases stored in the databases. The CALPHAD approach faces new challenges when applied to the development of multi-principal element alloys (HEAs and CCAs) due to large extrapolations from the thermodynamic models which may lead to inaccurate evaluations of the Gibbs energies. Many CALPHAD databases have been developed for traditional alloys so the full thermodynamic assessment of the Gibbs energies is usually given only for compositions enriched with one main element. In contrast, HEAs and CCAs lie in the central regions of compositional space, far from the known boundaries delimited by the binary and ternary subsystems. Consequently, the calculations in these vast composition spaces rely on the availability and quality of the descriptions of the lower order constituent systems and require large extrapolations from these lower order systems by assigning higher order interaction parameters, which are refined using available experimental data. Reliability and accuracy of the predictions increases when using more complete, experimentally verified, thermodynamic descriptions specially designed for CCA compositions (e.g., TCHEA and PanHEA special HEA databases developed by Thermo-Calc Software AB and CompuTherm LLC, respectively). A complete thermodynamic description (full assessment) of binary and ternary systems is generally sufficient to correctly describe thermodynamic properties of higher order (quaternary, quinary, etc.) systems because the probability of the occurrence of quaternary or higher order intermetallic phases is low and decreases rapidly with an increase in the number of components. Therefore, complete thermodynamic description of binary and ternary systems is considered to be sufficient for the correct prediction of thermodynamic properties of higher order systems by extrapolation from the lower order systems. Unfortunately, even the most developed thermodynamic databases lack in the complete thermodynamic description (full assessment) of all binary and, especially, ternary systems based on the elementary components present in the database. For example, the TCHEA3 database includes 26 elements that can form 325 binary and 2600 ternary systems, but only 294 binary and 136 ternary systems are assessed in the full range of composition and temperature. Therefore, complete thermodynamic description is currently unavailable for a huge number of quaternary (or higher order) alloy systems, which may have a potential practical interest. For those reasons, it is very important to know what the minimum level of the thermodynamic assessment of binary and ternary systems is to be sufficient to qualitatively predict equilibrium phases and phase transformations in higher order CCAs. 

The goal of this paper is to evaluate the quality of prediction of phases and phase compositions in a quaternary system at different levels of the assessment (thermodynamic description) of the respective ternary systems and a complete description of the respective binary systems. To achieve this goal, we used the current capabilities of a commercially specialized CALPHAD database and identified quaternary alloys systems that have a complete description of their binary and ternary sub-systems. We then calculated ternary and quaternary phase diagrams for these quaternary systems and considered them as “reference diagrams”, assuming that they are 100% accurate relative to known experimental data due to a complete thermodynamic description. A parallel coordinate plots method, which is briefly described in the following section, was used to display three-dimensional data related to the extent of phase regions in the quaternary compositional space. Finally, we artificially reduced the levels of the thermodynamic description of the ternary systems, calculated the ternary and quaternary phase diagrams using this incomplete description, and compared the calculations with the reference diagrams. This analysis allowed us to identify conditions when the databases with incomplete thermodynamic descriptions can still be used to reliably predict thermodynamic properties of quaternary systems.

## 2. Methods

**Figure 1:**
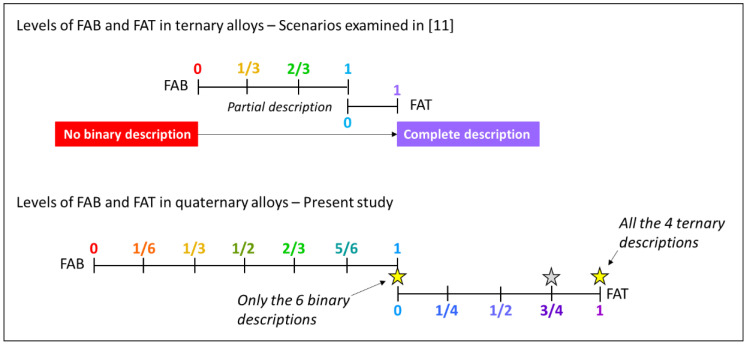
The possible values of the fraction of assessed binaries (FAB) and fraction of assessed ternaries (FAT) quantifying the fraction of binary/ternary descriptions assessed within a database for ternary and quaternary alloys. FAB = 1 and FAT = 0 represents partially assessed conditions, while FAB = 1 and FAT = 1 represents fully assessed conditions.

Senkov et al. [8,9] defined credibility criteria for CALPHAD calculations based on the fraction of fully thermodynamically assessed binary systems (FAB) and the fraction of fully assessed ternary systems (FAT) included in the database. By definition, a fully assessed system is one that has a complete thermodynamic description (of the respective elements, binary systems and ternary systems) within a given thermodynamic database allowing CALPHAD software to calculate thermodynamic properties for this system, which agree with the experimentally available data within the whole composition and temperature range. Using these theoretical credibility criteria, Wertz et al. [11] have recently demonstrated the importance of (a) the number of assessed constituent binary systems and (b) the compositional distance from them, on the accuracy of the predictions for ternary systems. Their study was carried out by comparing equilibrium calculations of the same ternary systems using eight different databases developed by CompuTherm LLC (Madison, WI, USA). Each of the studied ternary systems had complete thermodynamic descriptions (FAB = 1, FAT = 1) in at least one database, while other databases calculated the ternary space by extrapolation from binary descriptions with different levels of FAB and FAT = 0 (Figure 1a).

Here we expand a similar approach to higher order systems to evaluate the ability to predict quaternary phase diagrams. A quaternary system has 6 constituent binaries and 4 ternaries, so the possible FAB levels are 0, 1/6, 1/3, 1/2, 2/3, 5/6 or 1 for databases that include full thermodynamic descriptions for 0, 1, 2, 3, 4, 5 or 6 of the constituent binary systems, and the FAT values are 0, 1/4, 1/2, 3/4, or 1 for databases that include full thermodynamic descriptions for 0, 1, 2, 3, or 4 of the constituent ternary system (Figure 1b). The commercial database TCHEA3 from Thermo-Calc Software AB (Stockholm, Sweden) used in the present work includes 26 elements, 294 fully assessed binary and 136 fully-assessed ternary descriptions, providing few quaternary systems with FAB = 1 and FAT = 1 (e.g., Co-Cr-Fe-Ni and Co-Cu-Fe-Ni), and FAB = 1 and FAT = 3/4 (e.g., Al-Co-Ni-Ti, Al-Cu-Fe-Ni, Al-Cu-Fe-Ti, Al-Cu-Ni-Ti, Al-Mn-Ni-Ti, Al-Fe-Ni-Ti, Al-Ni-Si-Ti). Among them, we have selected three phase diagram templates for this study:

- Co-Cr-Fe-Ni (FAB = 1, FAT = 1), which consists of 3 disordered solid solutions (*fcc, bcc, hcp*) and one binary intermetallic having ternary solubility (σ);
- Co-Cu-Fe-Ni (FAB = 1, FAT = 1), which has only disordered solid solutions (*fcc* and *bcc* phases), with miscibility gap for *fcc*;
- Al-Co-Ni-Ti (FAB = 1, FAT = 3/4), which includes ordered and disordered solid solutions (*fcc*/L12 and *bcc*/B2) and several binary and ternary intermetallics.

TCHEA3 is an encrypted database which does not permit access to the values of the Gibbs free energy parameters of the phases. However, it is possible to fix the value of any parameter using the commands available in the Gibbs Energy System (GES) module implemented in Thermo-Calc® software. Using this command for all possible relevant parameters allows a comparison of the predictions for the reference systems, referred to as FAT=1 for Co-Cr-Fe-Ni and Co-Cu-Fe-Ni, and FAT = 3/4 for Al-Co-Ni-Ti (the Co-Ni-Ti ternary subcomponent is only partially assessed in TCHEA3), with the results obtained when (1) setting to zero all the ternary interaction parameters for all the relevant phases (solid solutions and a binary intermetallic having ternary solubility) but retaining ternary intermetallics and (2) additionally suspending all the ternary intermetallics. The scenarios (1) and (2) are referred to as FAT = 0 since they are equivalent to a database in which the ternary systems are not assessed for the three selected quaternary systems (the description of the quaternary relies only on the descriptions of the 6 constituent binaries). We have selected one comparison metric which is the extent of the compositional range of each phase throughout the 3D composition space. These data were retrieved from the phase point coordinates calculated at a fixed temperature for 12,341 equally spaced system points (2.5 at.% compositional steps) throughout the quaternary diagram. It is worth mentioning that our methodology uses the same commercial database for calculation of thermodynamic properties of a quaternary alloy at different levels of assessment of the respective ternary systems, which is achieved by artificial removal of the ternary interaction parameters. At the same time, the binary interaction parameters in the database containing the description of ternary systems can slightly differ from the published binary assessment to better reproduce the ternary phase diagram. However, these modifications are made in such a way that the calculated thermodynamic properties of the respective binary systems still agree with the accepted experimental data. Therefore, setting all ternary terms to zero (FAT=0) can still be treated as a condition with FAB = 1.

As previously discussed by Miracle and Senkov [12], and Wertz et al. [11], visualizing the extension and topology of binary and ternary terminal solid solution phase fields into a higher order dimensional composition space remains difficult because the properties of high dimensional objects diverge our two or three-dimensional intuition. An alternative approach to get an insight into the phase points (representative points of a phase, i.e., single-phase region and its boundaries) in high dimension is to use parallel coordinate plots [3,13]. In such a plot, the axes for the variables (e.g., composition of each alloying element and temperature) lie parallel to each other and span from the minimum to maximum value of each variable. The coordinates of a phase (i.e., phase points) or a phase field with two or more phases in equilibrium (i.e., system points) are visualized by segmented lines connecting each variable (molar composition and temperature), so the number of lines connecting the composition coordinates of a selected phase reflects the extent of that phase in the compositional space. It is then possible to evaluate the extent of a phase (or a phase field) in the n-dimensional space of a phase diagram as the ratio of the number of phase points over the number of system points. The ratio represents the (hyper)-volume fraction occupied by a phase in the n-dimensional composition space.

## 3. Results and Discussion

**Figure 2:**
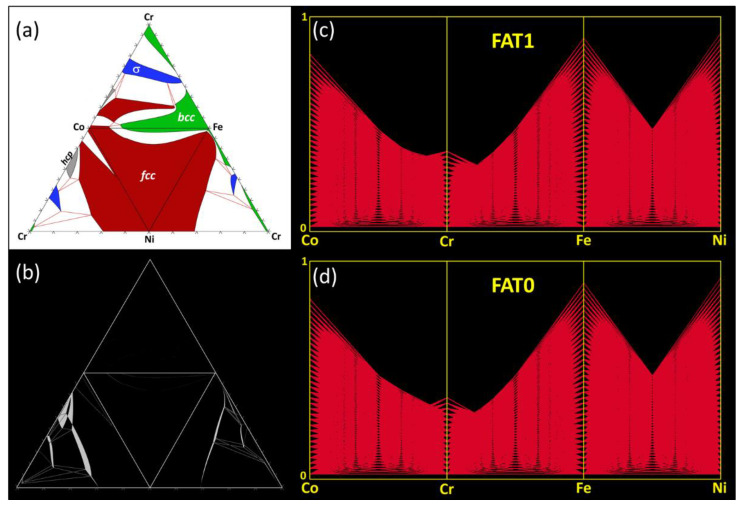
Co-Cr-Fe-Ni system: (**a**) Unfolded tetrahedral quaternary phase diagram showing the four isothermal sections of the ternary subsystems calculated at 800°C for FAT = 1, highlighting the single-phase fields. (**b**) Difference (appearing in white) between the extent of the various phase fields calculated for FAT = 1 and FAT = 0. (**c,d**) Parallel coordinate plots showing the predicted range of existence (4D compositional coordinates) of the *fcc* phase in the quaternary Co-Cu-Fe-Ni phase diagram at 800 °C for (c) FAT = 1 and (d) FAT = 0.

**Figure 3:**
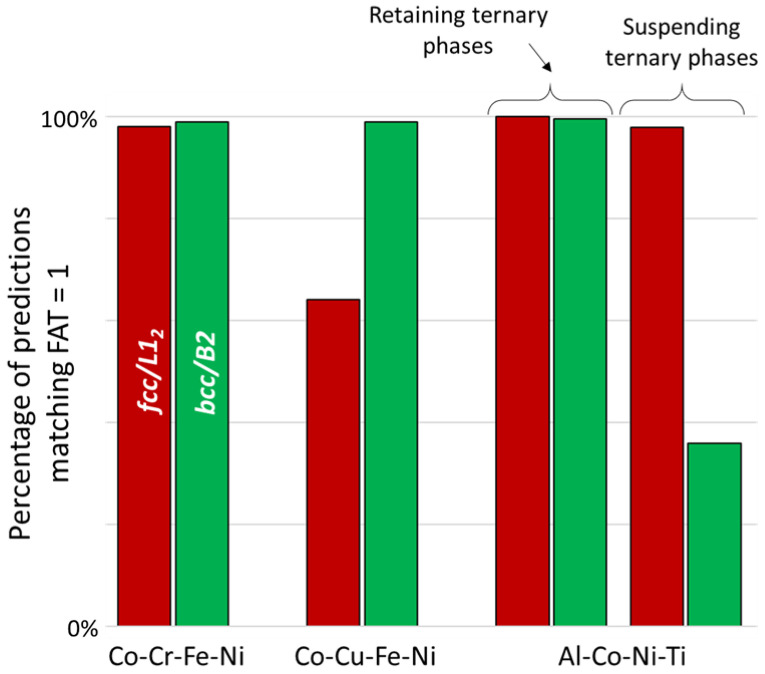
Percentage of predictions matching FAT = 1 for the Co-Cr-Fe-Ni and Co-Cu-Fe-Ni quaternary systems, for the *fcc*/L1_2_ and *bcc*/B2 phase points, respectively, when neglecting ternary interactions (FAT = 0). For the Al-Co-Ni-Ti system, the first condition corresponds to a special case of FAT = 0, when all ternary interactions are canceled but the ternary phases are artificially retained, while the second condition corresponds to the actual FAT = 0 condition, when both ternary interactions and ternary phases are suspended.

Figure 2a shows an unfolded tetrahedral quaternary phase diagram for the Co-Cr-Fe-Ni system showing the four isothermal sections of the ternary subsystems calculated at 800 °C under the condition FAT = 1. The single-phase fields are highlighted by red (*fcc*) green (*bcc*) gray (*hcp*) and blue (sigma) color codes, while the adjacent two- or three-phase regions are left un-colored (white). Figure 2b shows the difference between the extents of the phase fields in the subcomponent ternaries calculated for FAT = 1 and FAT = 0. The phase field regions where the calculations do not match are shown in white. It can be seen that the disagreements in the calculations appear mainly in the Co-Cr-Ni and Cr-Fe-Ni, while perfect match occurs in Co-Cr-Fe and Co-Fe-Ni. The disagreements of the FAT=1 and FAT=0 calculations for Co-Cr-Ni mainly appear in the regions associated with *hcp* and δ phases and at phase-field boundaries. In Cr-Fe-Ni, these entirely occur at the phase-field boundaries. In Figure 2c–d, the parallel coordinate plots show the *fcc* phase points in the Co-Cr-Fe-Ni quaternary system for FAT = 1 and FAT = 0, respectively. There is no significant difference between both predictions. As summarized in Figure 3, the phase points within the *fcc* and *bcc* phase fields calculated using FAT = 0 match 98% of the FAT = 1 predictions. These results suggest that the phase equilibria and locations of the phase boundaries (solubility limits) delimiting the single solid solution regions in the Co-Cr-Fe-Ni quaternary diagram are weakly sensitive to the number of assessed ternary subsystems. In other words, ternary contributions are negligible, and the accuracy remains when extrapolating the Co-Cr-Fe-Ni quaternary system exclusively from the six fully-assessed binary subsystems. Correct prediction of miscibility gap and/or intermetallic phases may however require more complete description of ternary interactions.

**Figure 4:**
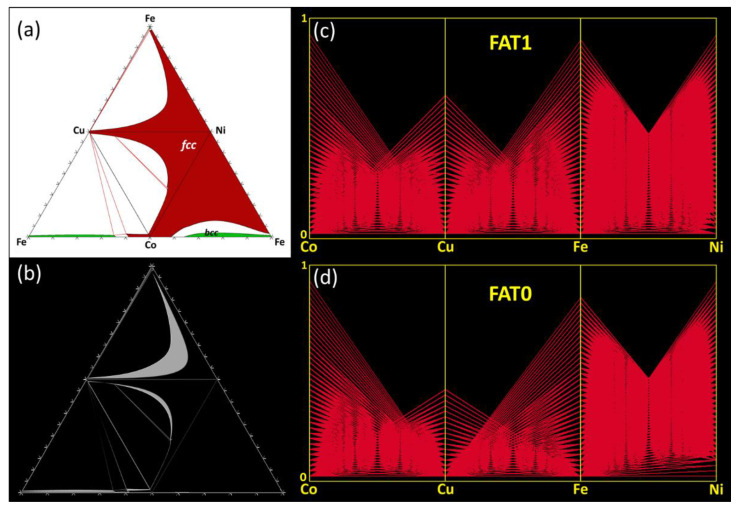
Co-Cu-Fe-Ni system: (**a**) Unfolded tetrahedral quaternary phase diagram showing the four isothermal sections of the ternary subsystems calculated at 800°C for FAT = 1, highlighting the single-phase fields. (**b**) Difference (appearing in white) between the extent of the various phase fields calculated for FAT = 1 and FAT = 0. Parallel coordinate plots showing the predicted range of existence (4D compositional coordinates) of the *fcc* phase in the quaternary Co-Cu-Fe-Ni phase diagram at 800 °C for (**c**) FAT = 1 and (**d**) FAT = 0.

The effects from canceling ternary interactions are slightly different for the Co-Cu-Fe-Ni (Figure 4). This system contains disordered *fcc* and *bcc* phases and no intermetallic phases (Figure 4a). The calculations with FAT = 0 correctly predict the presence and location of these phases. Perfect match of FAT = 0 and FAT = 1 calculations occurs for the Co-Fe-Ni ternary, similar to the previous case with the Co-Cr-Fe-Ni system. However, the main disagreement between the FAT = 0 and FAT = 1 calculations arises from incorrect predictions of the miscibility gap boundaries for the high-temperature fcc phase in the Co-Cu-Ni and Cu-Fe-Ni ternary subcomponents (Figure 4b) and in the quaternary volume (Figure 4c,d). The absence of ternary interactions incorrectly shifts the miscibility gap boundaries toward higher concentrations of Ni, while the presence of these Ni-associated ternary interactions expands the high-temperature *fcc* phase to higher concentrations of Cu and Fe or Co and Cu (Figure 4b–d). Due to this, only 63% of the *fcc* phase points calculated from FAT = 0 match the FAT = 1 calculations, while almost 100% agreement occurs for the bcc phase fields (Figure 3).

**Figure 5:**
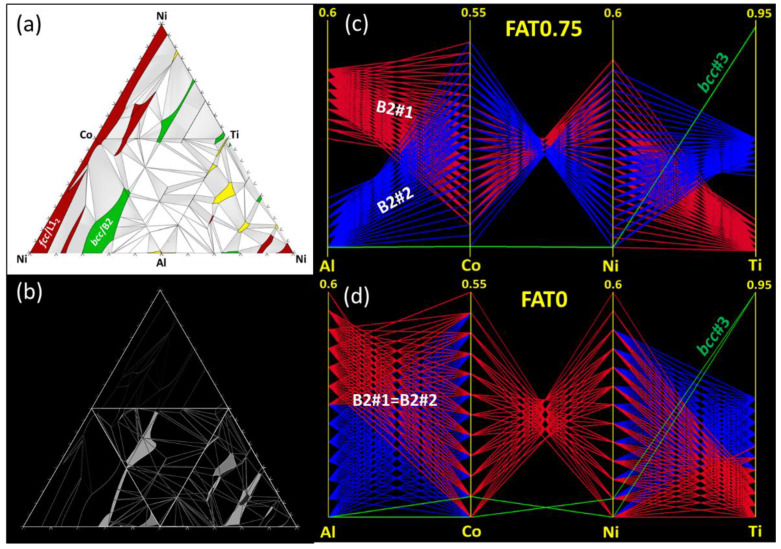
Al-Co-Ni-Ti system: (**a**) Unfolded tetrahedral quaternary phase diagram showing the four isothermal sections of the ternary subsystems calculated at 800°C (FAT = 3/4), highlighting the *single-*phase fields. In addition to the solid solution phases, the quaternary system includes several ordered solid solutions (L1_2_, B2), binary intermetallics (Al_5_Co_2_, Al_3_Co, Al_13_Co_4_, Al_9_Co_2_, Al_3_Ni, Al_3_Ni_2_, BCT, Al_2_Ti, AlTi, AlTi_3_, Ni_3_Ti, NiTi_2_, Laves-C15 and Laves-C16) and several ternary intermetallics (H_L2_1_, Laves_C14). (**b**) Difference (appearing in white) between the extent of the various phase fields calculated for FAT = 3/4 and FAT = 0 (the case when all ternary intermetallics are suspended, see Figure 3). Parallel coordinate plots showing the predicted range of existence (4D compositional coordinates) of the *bcc* phase in the quaternary Al-Co-Ni-Ti phase diagram at 800 °C for (**c**) FAT = 3/4 and (**d**) FAT = 0.

The discrepancies between predictions performed based on ternary descriptions and those without are even more severe for the Al-Co-Ni-Ti system (Figure 5). In addition to the disordered fcc and bcc solid solutions, this system also contains several ordered (L1_2_, B2) binary intermetallic (Al_5_Co_2_, Al_3_Co, Al_13_Co_4_, Al_9_Co_2_, Al_3_Ni, Al_3_Ni_2_, BCT, Al_2_Ti, AlTi, AlTi_3_, Ni_3_Ti, (Co,Ni)Ti_2_, Laves-C15 and Laves-C16) and several ternary intermetallic (H_L2_1_, Laves_C14) phases (Figure 5a). The presence of many different phase field regions in this quaternary system results in high density of boundaries between these regions, where the main disagreements between FAT = 0 and FAT = 1 calculations occur. In addition, FAT = 0 calculations cannot predict composition regions for ternary intermetallic phases. Instead, they fill these regions with available binary phases (mainly the bcc and B2 phases). This can be well visualized by comparing the bcc and B2 phase points represented in parallel coordinate plots for FAT = 3/4 (Figure 5c) to FAB = 0 (Figure 5d).

The different sensitivities to FAT of the calculated phase diagrams for Co-Cr-Fe-Ni, Co-Cu-Fe-Ni and Al-Co-Ni-Ti systems result from the different thermodynamic properties and topologies of these systems. In all the cases, the number of solid solution phases and solid-solution phase identifications match perfectly between FAT = 1 (or ¾) and FAT = 0 calculations. The main differences in the calculations occur at or near the boundaries between different phase fields. Therefore, higher density of these boundaries should result in lower accuracy of the FAT = 0 calculations. This observation is in agreement with earlier results by Wertz et al. [11]. The presence of ternary phases in the ternary systems, which the evaluated higher order system contains, will result in incorrect FAT = 0 calculations, as the extrapolations from the binary interactions cannot predict formation of ternary phases. Unfortunately, the locations of the phase boundaries and possible presence of ternary phases are not known when evaluating novel composition space. Therefore, a higher credibility database is still preferable, while the calculations using lower credibility databases may be questionable and require experimental verification.

The Co-Cr-Fe, Co-Cr-Ni, Co-Fe-Ni and Cr-Fe-Ni behave like near-ideal mixtures of the constituent elements so the Co-Cr-Fe-Ni quaternary does too, which results in the stabilization of the disordered solid solutions and the absence of ternary intermetallic phases. In such cases, neglecting ternary interaction parameters when considering FAT = 0 predictions gives approximatively the same results as for FAT = 1 because their contributions to the Gibbs free energy are also negligible. The Co-Cu-Fe-Ni quaternary system deviates to the near-ideal behavior due to a tendency for the demixing of the Cu-Co and Cu-Fe constituent binaries. In this case, the extent of the miscibility gap is sensitive to the ternary interactions and neglecting them causes incorrect predictions of the phase boundaries involving the solid solutions with the miscibility gap.

In contrast, the Al-Co-Ni-Ti system exhibits several ordered solid solutions and binary and ternary intermetallics. In this case, the most significant factor controlling the location of the phase boundaries arises from the phase assemblage, i.e., the presence and proximity of ternary intermetallics, whereas the removal of the ternary interaction parameters does not change significantly the number of phase points matching the reference predictions when the ternary phases are maintained (Figure 3). This sensitivity depends upon the neighboring phase regions. In particular, the assemblage around the *fcc* and L1_2_ regions consists of various binary and ternary phase fields involving B2, Ni_3_Ti and not containing ternary intermetallics, so the extent of the *fcc* region is not constrained when ternary intermetallics are removed. In consequence, using an incomplete database (FAT = 0) does not substantially affect the composition and boundaries of the *fcc* and L1_2_ phases. On the other hand, *bcc* and B2 regions are surrounded by several ternary intermetallics (γ and H_L2_1_), so neglecting them (FAT = 0) shifts significantly the *bcc* and B2 composition and boundaries, thus providing significant disagreement between FAT = 3/4 and FAT = 0 calculations (Figure 3).

## 4. Conclusions

The ability of a commercial CALPHAD database for high entropy alloys to predict quaternary phase diagrams has been evaluated through comparisons of predictions made from full binary system descriptions with the results from full ternary system descriptions. This point is of prime interest because even the most comprehensive databases (such as TCHEA3 from Thermo-Calc) include a limited number (about 5%) of fully assessed ternary systems. This entails that quaternary and higher order predictions are systematically obtained from incomplete thermodynamic descriptions and rely almost entirely on extrapolations from binary subsystem descriptions. The analysis has led to the following conclusions.

(1) The quality of CALPHAD calculations for 4-component alloys mainly depends on the ability to correctly describe (a) all the phases present in binary and ternary sub-systems, including novel ternary phases, and on (b) positions (composition and temperature) of boundaries between different phase-field regions.
(2) Direct extrapolation from binaries to quaternary (i.e., without ternary descriptions) systems may be acceptable only when binary miscibility gaps are not present, binary intermetallic phases are not present or present in a few quantities (i.e., when the system has low density of phase boundaries), and ternary intermetallic phases are not present.
(3) When ternary phases are present but not included in the thermodynamic descriptions, the prediction accuracy of the FAT = 0 calculations will be strongly affected only for neighboring phase regions.

Considering that quaternary and higher order interactions are negligibly weak, and the formation of novel quaternary intermetallics very unlikely, the present conclusions for quaternary alloys can be extended to higher order systems. Because the locations of the phase boundaries and possibility of formation of ternary phases are not known when evaluating novel composition space, a higher credibility database is still preferable, while the calculations using lower credibility databases may be questionable and require additional experimental verification.
